# Nonoperative Management of Acute Appendicitis in the Geriatric Population: A Review

**DOI:** 10.7759/cureus.73331

**Published:** 2024-11-09

**Authors:** Amir Farah, Anna Tatakis, Amir Obeid, Sa'd Sayida

**Affiliations:** 1 Surgery, Medical College of Wisconsin, Milwaukee, USA; 2 General Surgery, Medical College of Wisconsin, Milwaukee, USA; 3 General Surgery, HaEmek Medical Center, Afula, ISR; 4 General Surgery, Edinburgh Medical Missionary Society Nazareth Hospital, Nazareth, ISR

**Keywords:** acute care surgery, appendectomy, appendicitis, geriatrics, nonoperative management

## Abstract

Appendicitis remains a common cause of acute abdominal pain, particularly challenging to manage in elderly patients due to age-related declines in physiological reserve and the presence of comorbidities. While appendectomy has traditionally been the standard treatment, nonoperative management (NOM) using antibiotics has emerged as a potential alternative for elderly patients, especially those considered frail or at high surgical risk. This review explores the efficacy and outcomes of NOM compared to surgery in this population, with a focus on recurrence rates, postoperative complications, and the impact of frailty on treatment decisions.

Recent studies highlight both the benefits and limitations of NOM. While it can effectively manage uncomplicated appendicitis in the short term, recurrence rates are significantly higher in elderly patients, often necessitating delayed surgery, which can lead to worse outcomes. Research also shows that frail patients face elevated risks of complications, including mortality, whether treated surgically or nonoperatively. The review underscores the importance of careful patient selection, close monitoring, and individualized treatment approaches when considering NOM for elderly patients with appendicitis. Long-term risks such as recurrence, antibiotic resistance, and complications further complicate the decision-making process.

## Introduction and background

Appendicitis remains a common cause of acute abdominal pain that often requires emergency intervention. In younger, healthier individuals, appendectomy is generally considered safe and effective. However, elderly patients with multiple comorbidities present a different set of challenges. Age-related declines in physiological reserve, along with conditions like diabetes, cardiovascular disease, and chronic kidney disease, elevate surgical risks [1]. These patients are at increased risk for postoperative complications, including infection, prolonged recovery, and even death. Moreover, atypical presentations of appendicitis in the elderly, such as delayed or unclear symptoms, can lead to delays in diagnosis and treatment, often resulting in higher rates of perforation and abscess formation [2].

For decades, appendectomy has been the definitive treatment for appendicitis due to fears of perforation and peritonitis. However, in recent years, nonoperative management (NOM), which involves the use of antibiotics instead of surgery, has gained traction. The rationale behind NOM is to avoid the risks associated with surgery, particularly for frail elderly patients who may not tolerate anesthesia and surgical intervention well [3]. This shift is partly due to research indicating that many cases of uncomplicated appendicitis can be effectively treated with antibiotics, thus avoiding the need for immediate surgery.

Elderly patients, defined as individuals aged 65 and older, face a significantly higher risk of postoperative complications following appendectomy, including respiratory failure, wound infections, and thromboembolic events [4]. This increased risk has led to an exploration of NOM as a safer alternative in this population, especially for those classified as frail - a clinical syndrome characterized by diminished physiological reserves and increased vulnerability to external stressors [5]. This review aims to explore the role of NOM in the management of appendicitis in elderly patients with multiple comorbidities, comparing its efficacy to appendectomy, examining recurrence rates, and discussing factors influencing the success of NOM, particularly the role of frailty.

## Review

The shift toward nonoperative management

Historically, appendectomy was performed in nearly all cases of appendicitis due to the belief that untreated appendicitis would inevitably progress to perforation. However, this view has been challenged by studies demonstrating that antibiotics alone can successfully treat uncomplicated appendicitis in a substantial proportion of patients. One of the pivotal studies initiating this shift was the APPAC trial, which found that antibiotics successfully treated appendicitis in 73% of patients over a five-year follow-up period [4]. Although appendectomy remains essential for complicated appendicitis, NOM has emerged as a viable alternative for elderly patients who are at higher risk for surgical complications.

The rise in NOM has largely been driven by the desire to minimize the surgical risks that elderly patients face. Surgery in the elderly is associated with significantly higher morbidity and mortality rates due to their diminished physiological reserves and the presence of comorbidities [6].

Horn et al. conducted a comprehensive analysis of appendicitis management trends in the United States, observing an increasing use of NOM in elderly patients with multiple comorbidities. Their findings revealed that while NOM is associated with decreased hospital charges, it also significantly increases the risk of mortality in this demographic. This underscores the importance of careful patient selection when considering NOM, as elderly patients with greater medical complexity may be at heightened risk for poor outcomes if treated nonoperatively. These results suggest that, for some frail or elderly patients, early surgical intervention may still offer a better prognosis, even with the associated surgical risks [2].

Mason argued that surgery may not be necessary for all appendicitis cases, especially in patients presenting with uncomplicated forms of the condition. His study suggested that a more conservative approach, involving antibiotics to resolve inflammation, could spare patients from surgical risks, including infections, anesthesia-related complications, and prolonged recovery times [1]. This view was supported by Vons et al., who demonstrated that antibiotics could effectively treat uncomplicated appendicitis, avoiding the need for surgery in many cases. However, their findings also indicated that patients treated with antibiotics had a higher incidence of postintervention complications such as peritonitis (8%) compared to those who underwent surgery (2%) and that 29% of patients in the antibiotic group required surgery within a year due to recurrence. This highlights the limitations of NOM, particularly in its ability to prevent long-term complications [3].

Efficacy of nonoperative management in elderly patients

Despite its potential benefits, the efficacy of NOM in elderly patients remains a topic of debate. Several studies have demonstrated that antibiotics can successfully treat appendicitis in elderly patients, but there are concerns about the long-term success of this approach. Salminen et al. provided long-term follow-up data from the APPAC trial, showing that while antibiotics initially resolved appendicitis in many cases, the recurrence rate reached 39.1% over a five-year period. Approximately 30% of patients required surgery within the first year, indicating that while NOM can be effective in the short term, the long-term risk of recurrence is significant, especially in frail elderly patients who may not tolerate delayed surgery as well [4].

Meier et al. found that elderly patients treated with NOM were more likely to experience recurrent appendicitis than younger patients. Their study reported that while antibiotics were initially effective, the risk of recurrence was significantly higher in elderly patients, with many requiring surgeries within a year of their initial treatment [6]. This finding aligns with Chehab et al., who reported that elderly patients experiencing recurrence after NOM had significantly higher mortality rates than those who underwent appendectomy upfront [5]. These studies suggest that while NOM may be effective in the short term, it carries significant long-term risks, particularly for elderly patients.

Risks of recurrence and complications

The primary risk associated with NOM is the potential for recurrence. Studies have consistently shown that recurrence rates are higher in elderly patients treated nonoperatively than in younger patients. In a retrospective analysis, Lie et al. found that the recurrence rate for elderly patients treated with NOM ranged from 20% to 30% within the first year. In particular, the authors identified the presence of an appendicolith on imaging as a strong predictor of recurrence. Patients with appendicoliths were significantly more likely to experience recurrent appendicitis within six months, suggesting that careful radiographic assessment should be a key factor in determining the suitability of NOM for elderly patients [7].

Dhillon et al. found that elderly patients who required delayed surgery after failed NOM had worse outcomes than those who underwent immediate appendectomy. Their study showed that elderly patients who underwent delayed surgery had higher rates of postoperative complications, including sepsis, organ failure, and prolonged hospital stays [8]. Moreover, the study revealed that nearly half of elderly patients diagnosed preoperatively with uncomplicated appendicitis were found to have complicated appendicitis upon pathological examination. This discrepancy between preoperative assessment and actual pathology suggests that NOM may not be appropriate for many elderly patients, as it increases the risk of undiagnosed complications that could worsen outcomes if surgery is delayed. These findings underscore the importance of patient selection when considering NOM for elderly patients with appendicitis. Patients with a high likelihood of recurrence may benefit more from immediate surgery, even if they are considered Rx for operative intervention.

Additionally, Joseph et al. reported that frail elderly patients who experienced recurrence after NOM were more likely to develop serious complications, such as abscess formation and sepsis [9]. These complications significantly increase the risk of mortality in elderly patients, emphasizing the importance of early intervention in cases of recurrent appendicitis. While NOM may reduce the immediate risks of surgery, it is not without its own risks, particularly for elderly patients who are already vulnerable to complications due to frailty and comorbidities.

The role of frailty in acute care surgical treatment decisions

Frailty is a key factor in determining the appropriateness of NOM in elderly patients with appendicitis. Frailty is defined as a clinical syndrome characterized by decreased physiological reserves and increased vulnerability to stressors such as surgery and infection. Frail patients are more likely to experience adverse outcomes following both surgery and NOM, making treatment decisions particularly challenging in this population [10]. Joseph et al. (2017) found that frail elderly patients were significantly more likely to experience in-hospital complications (33.4%) compared to non-frail patients (12%), and also had higher rates of trauma-related readmissions and mortality [9]. This highlights the importance of a personalized approach to treatment and assessing frailty in elderly patients when considering NOM, as frail individuals may be less capable of handling the delayed interventions required if NOM fails.

Ashbrook et al. emphasized that frailty, rather than age alone, should be the primary determinant of treatment decisions for elderly patients with appendicitis. In their study, they demonstrated that frail patients treated with NOM had nearly three times the risk of in-hospital mortality compared to those who underwent immediate appendectomy [10]. This finding highlights the significant risks associated with NOM in frail elderly patients, suggesting that immediate surgery may be a safer option in this vulnerable population and that frail patients may not benefit from NOM as much as their non-frail counterparts, as they are more likely to experience complications and may not tolerate delayed surgery required if NOM fails.

Comparative outcomes: Surgery vs. nonoperative management

Several studies have compared the outcomes of NOM and surgery in elderly patients, with mixed results. Meier et al. found that while NOM was associated with fewer immediate complications, it also resulted in higher rates of recurrence and longer hospital stays in the long term [6]. Meier et al. further noted that NOM reduced the risk of complications by 3.72% but increased mortality rates by 1.82%. This suggests that while NOM may initially appear to reduce the burden of complications, the increased mortality risk in elderly patients indicates that NOM is not without significant dangers, especially for frail patients [6]. Their study reported that elderly patients who underwent delayed surgery after failed NOM had significantly higher mortality rates and worse postoperative outcomes compared to those who underwent immediate appendectomy.

Similarly, Dhillon et al. found that elderly patients who underwent surgery after failed NOM had worse outcomes than those who underwent immediate appendectomy, including higher rates of sepsis, organ failure, and prolonged hospital stays [8]. The study also emphasized that nearly half of the elderly patients diagnosed preoperatively with uncomplicated appendicitis were found to have complicated appendicitis during surgery [8]. This finding raises further concerns about relying on NOM in the elderly population, as it may delay necessary surgical intervention for patients who are already at a higher risk of complications. These findings suggest that while NOM may be appropriate for select patients, it carries significant risks if not closely monitored. Elderly patients who are likely to experience recurrence may benefit more from immediate surgery, despite the inherent risks associated with operative intervention.

On the other hand, Vons et al. suggest that NOM can be a viable alternative to surgery for uncomplicated appendicitis, particularly in elderly patients with frailty or other comorbidities [3]. However, the authors cautioned that while antibiotics were effective in treating uncomplicated appendicitis, the recurrence rate remained high (29% within one year). Additionally, patients treated with antibiotics were at a greater risk of postintervention complications, such as peritonitis (8% in the NOM group versus 2% in the surgery group). This reinforces the idea that careful patient selection and close monitoring are crucial when opting for NOM [3]. The decision to pursue NOM must be individualized based on the patient’s overall health status, frailty, and ability to tolerate surgery in the event of recurrence.

Long-term considerations for nonoperative management

The long-term outcomes of NOM in elderly patients remain an area of concern, particularly regarding recurrence and delayed complications. While antibiotics can effectively treat appendicitis in the short term, the risk of recurrence and the need for delayed surgery often outweigh the benefits of NOM in the long term, especially in frail elderly patients. Salminen et al. found that while NOM was initially successful, recurrence rates increased over time, with approximately 30% of patients requiring surgery within the first year of treatment, and a cumulative recurrence rate of 39.1% over five years [4]. This emphasizes the need for long-term follow-up, as recurrence may occur months or years after the initial episode, making NOM less appealing for elderly patients who may not tolerate delayed surgery well.

Vons et al. similarly observed that while antibiotics were effective in managing uncomplicated appendicitis initially, nearly 29% of patients required surgery within a year due to recurrence [3]. They also noted a higher risk of complications, such as peritonitis, in the NOM group (8% vs. 2% for surgical patients) [3]. These findings suggest that, despite short-term benefits, NOM carries significant risks of recurrence and complications, particularly for elderly patients with comorbidities.

In addition to concerns about recurrence, the long-term use of antibiotics in elderly patients raises issues of antibiotic resistance and adverse drug reactions. Khalil et al. emphasized that elderly patients, who often take multiple medications for other conditions, are at increased risk of antibiotic resistance, which could diminish the effectiveness of future treatments [11]. Moreover, prolonged use of antibiotics can lead to Clostridium difficile infections, a serious complication that is more prevalent in older adults and can significantly increase mortality rates.

Finally, Meier et al. pointed out that while NOM may reduce the immediate risks of surgery, the long-term outcomes, including increased recurrence and mortality rates, may outweigh these short-term benefits [6]. Their findings highlight that for elderly patients, especially those who are frail, the long-term risks associated with NOM-such as delayed surgery and complications-often make it a less favorable option compared to immediate surgery. These studies collectively highlight that while NOM may initially reduce the burden of surgery in elderly patients, its long-term risks, particularly in frail individuals, suggest that careful patient selection and long-term monitoring are crucial. For many elderly patients, especially those at high risk of recurrence or complications, surgery may ultimately provide better long-term outcomes.

Discussion

The use of NOM in elderly patients with appendicitis presents a complex decision-making process, balancing the immediate risks of surgery with the potential for long-term complications. One critical factor in this decision is the patient's frailty status. Studies such as those by Meier et al. and Salminen et al. show that frail patients face increased mortality and long-term complications, even if NOM initially reduces surgical risks [4,6]. This highlights the need for individualized treatment decisions that account for both the patient’s overall health and the risks of delayed intervention.

Patient selection remains a cornerstone for the successful implementation of NOM in elderly patients. Research, including that by Vons et al. and Dhillon et al., underscores the dangers of underestimating the complexity of appendicitis in elderly patients, particularly the risk of recurrence and complications [3,8]. These studies show that undetected complications, such as peritonitis, can arise even in cases initially deemed uncomplicated, making close monitoring essential for patients managed nonoperatively. The presence of an appendicolith, as highlighted by Lie et al., serves as a strong predictor of recurrence, reinforcing the importance of careful imaging and clinical assessment before committing to NOM [7].

While NOM offers the potential to reduce immediate surgical risks, its long-term efficacy is less clear. Studies by Salminen et al. and Meier et al. have shown that recurrence rates can be substantial, with up to 30-40% of patients requiring delayed surgery after an initial course of antibiotics [4,6]. This concern is particularly relevant in frail elderly patients who may be at increased risk of morbidity and mortality from delayed surgical interventions. In this context, the results of the meta-analysis by Sallinen et al. add further weight to the argument that while antibiotics can be effective in the short term, the cumulative recurrence rates, especially over a five-year period, raise concerns about the long-term sustainability of NOM as a definitive treatment strategy for elderly patients [12].

Another significant concern with prolonged or recurrent antibiotic treatment is the risk of developing antibiotic resistance or complications from long-term antibiotic use, including Clostridium difficile infections. As noted by Khalil et al., elderly patients are especially vulnerable to these risks, given their frequent use of multiple medications for chronic conditions [11]. Future research should address the impact of prolonged antibiotic use and the growing threat of antimicrobial resistance, particularly in frail populations where treatment options are already limited.

In addition to the risk of recurrence, studies have raised concerns about the accuracy of preoperative diagnostics in elderly patients undergoing NOM. Dhillon et al. found that nearly half of elderly patients diagnosed with uncomplicated appendicitis were found to have complicated appendicitis at the time of surgery [8]. This highlights the limitations of relying solely on clinical and imaging criteria when determining whether an elderly patient is a good candidate for NOM.

However, the pilot randomized controlled trial by Talan et al. suggests that outpatient antibiotic management for appendicitis is feasible, which could potentially reduce hospital stays and associated costs for select patients [13]. The trial emphasizes that outpatient NOM could be a practical alternative for uncomplicated appendicitis, provided that patients are carefully selected and rigorously monitored to manage the risks of recurrence and complications.

On the other hand, studies like that of Frazee et al. advocate for the consideration of outpatient laparoscopic appendectomy as a standard of care for uncomplicated appendicitis. Their research points to the efficacy and safety of laparoscopic surgery even in outpatient settings, which may offer a middle ground for elderly patients who are at risk from prolonged antibiotic therapy or delayed interventions. Frazee et al. argue that laparoscopic appendectomy can reduce the risk of recurrent appendicitis and avoid the complications associated with delayed surgical treatment while minimizing hospital stays and recovery times for patients, even in older populations [14]. This finding suggests that in certain cases, particularly for patients with frailty or comorbidities that complicate recovery from severe infections or prolonged antibiotic use, outpatient surgery may offer a safer long-term solution compared to NOM.

Overall, the existing literature suggests that while NOM may offer a viable short-term alternative to surgery for select elderly patients, its long-term efficacy remains uncertain. The risk of recurrence, complications from antibiotic therapy, and the challenges of patient selection continue to make NOM a less favorable option for many elderly patients, particularly those who are frail. Careful patient selection, close monitoring, and the availability of rapid surgical intervention remain crucial to optimizing outcomes. Furthermore, the possibility of outpatient laparoscopic surgery, as proposed by Frazee et al., presents an intriguing option that deserves further exploration in future research.

## Conclusions

NOM presents a viable alternative to surgery for elderly patients with appendicitis, particularly those with significant comorbidities or frailty, offering the potential to avoid the immediate risks of surgical intervention. However, the long-term success of NOM is limited by high recurrence rates and complications, such as delayed surgery and increased mortality. Given these risks, treatment decisions should be individualized, considering the patient's overall health, frailty, and ability to tolerate both surgical and nonoperative approaches. Close monitoring and the option for timely surgical intervention remain crucial for optimizing patient outcomes. Ultimately, while NOM may be beneficial for select patients, surgery remains a more definitive treatment, particularly for those at higher risk of recurrence or complications.
